# Musical Novices Are Unable to Judge Musical Quality from Brief Video Clips: A Failed Replication of Tsay (2014)

**DOI:** 10.3390/vision6040065

**Published:** 2022-11-09

**Authors:** Jonathan M. P. Wilbiks, Sung Min Yi

**Affiliations:** Department of Psychology, University of New Brunswick Saint John, 100 Tucker Park Road, Saint John, NB E2L 4L5, Canada

**Keywords:** musical performance, thin slicing, auditory perception, visual perception

## Abstract

Research focusing on “thin slicing” suggests in making judgements of others’ moods, personality traits, and relationships, we are able to make relatively reliable decisions based on a small amount of information. In some instances, this can be done in a matter of a few seconds. A similar result was found with regard to the judgement of musical quality of ensemble performances by Tsay (2014), wherein musical novices were able to reliably choose the winner of a music competition based on the visual information only (but not auditory or audiovisual information). Tsay argues that this occurs due to a lack of auditory expertise in musical novices, and that they are able to extract quality information based on visual movements with more accuracy. As part of the SCORE project (OSF, 2021), we conducted a direct replication of Tsay (2014). Findings showed that musical novices were unable to judge musical quality at a level greater than chance, and this result held for auditory, visual, and audiovisual presentation. This suggests that 6 s is not a sufficient amount of time for novices to judge the relative quality of musical performance, regardless of the modality in which they were presented.

## 1. Introduction

In making assessments about others’ moods, personality traits, and relationships, we are able to make relatively reliable decisions based on a small amount of information [1,2]. This ability has been deemed ‘thin slicing’ and involves using cognitive and social heuristics to assess such traits and has been researched extensively. Additional research has also sought to examine this phenomenon in fields such as assessment of performance.

Tsay [3] investigated the effect of differing modalities of cues and leaders (conductors) in the judgement of musical performances while auditory cues are perceived to be the most important cue in music. Musical novices selected winners from performances by three competition finalists in sound-only, visual-only, or audiovisual clips and reported what cues mattered the most when judging [3]. Although all groups of participants in all experiments (musical novices and professionals) reported that sound is the most important information when evaluating music, visual-only conditions always exhibited the best accuracy when selecting winners while sound-only and audiovisual conditions had the worst, or below chance level accuracy. Tsay notes that judgment of winners is mostly based on visual rather than auditory cues and that sound might actually distract people from selecting actual winners. These results indicate that people, even musicians as well as non-musicians, appear to overweight visual information in their evaluation of music performances, so it is strongly encouraged that musicians and music adjudicators should focus more on the specific ways that visual cues affect music. 

An important task in science is the ability to evaluate the credibility of research claims, based on both face validity as well as statistical reliability. Individual research papers are not stand-alone pieces of evidence, but rather contributions to the corpus of a field at large, and it is important to be able to assess which pieces of evidence are more or less valuable to that field. The Systematizing Confidence in Open Research and Evidence (SCORE; [4]) project is an endeavour seeking to accomplish two goals–first, to assess individual research claims from studies in the social sciences over time, and second, to consider the elements in specific studies that may make the evidence coming from them more or less reliable to the field. The current study involves a replication of a study from Tsay [3], in which the authors assessed the ability of non-musicians to evaluate musical performances based on very short extracts of the performances. In this case, the claim from the original paper by Tsay suggesting that it is possible to successfully assess musical performances from a 6-s clip could have negative repercussions on evaluation of such performances. For example, when allowing somebody to audition for an orchestra of for a place in a music performance program, one may choose to make a decision based on a very brief piece of an audition. However, if the effect reported by Tsay is spurious, we may be doing a disservice to performers by not assessing their full performance. 

### 1.1. Musical Elements

The relative impact of visual and auditory cues has been studied by Thompson, Graham, and Russo [5]. They explored the influence of audio-visual integration in listeners’ perception of music and how visual aspects affect the communication between performers and listeners. To test this, Thompson et al. [5] divided participants into either audio-only or audiovisual groups and asked them to evaluate the level of disharmony of the performances that had either strong sense of dissonance or neutral facial expressions. Ratings were significantly higher in visually dissonant mode than neutral expressivity for audiovisual group, but this was not seen in audio-only group, which resulted from listeners integrating visual with auditory aspects of performance to form an audiovisual mental representation of music and this representation is not entirely predictable from the auditory input alone [5]. These findings indicate that facial expressions and gestures hugely contribute to visual cues in music, which enhance audience’s experience and interpretation of the music performances.

Siminoski [6] tested if audiences’ understanding of musical performances is affected by the two main cues in music—auditory and visual—by making clarinetist and pianist duets in four performer conditions: (1) normal setting, (2) no visual with full audio-only feedback, (3) full visual with partial auditory feedback, and (4) no visual but partial auditory feedback and asking participants to judge on expression, unity, and their subjective likeability of the performances that were presented in either audio-only, visual-only, or audiovisual clips. Normal performance setting ended up with highest ratings across all aspects and types of stimuli, and audio-only condition had no differences in ratings across the performer conditions while visual-only and audiovisual had significantly more differences [6]. 

Pope [7] examined how performance quality in both auditory and visual components and evaluators’ music experience shape music judgments by varying the quality of performance in audio and video (good or poor) and assigning participants into one of the four conditions: audio-only, video-only, good video + good or poor audio, or poor video + good or poor audio to rate on their musical aspects. Good quality performances gave significantly higher ratings than poor quality for all evaluation aspects, for almost all evaluation aspects, good video + good or poor condition had higher ratings than the other three conditions [7]. The authors argue that this is possibly because of string orchestras’ lessening of the influence of aural deficiencies in their performances by demonstrating good visual presentations, which shows that visual aspects in performance impact the judgments of many musical characteristics although the performances are primarily auditory. 

Tsay (2014) published another study on the importance of visual and auditory stimuli when evaluating music performances, and questioned the common belief that sound is the most reliable source of information when judging music [8]. Tsay found that although the participants reported that sound matters most to their judgments, both musical novices and experts successfully identified the winners of music competitions through silent videos (visual-only) but were unable to do so with audio-only or even with audiovisual recordings [8]. This suggests that the influence of visual cues is not affected in the experience in music, and that visual cues are likely overweighted when they are neither valued nor recognized stimuli when judging music, which may be because of the pressures that constrain our cognitive resources that lead to a visual dependence. 

However, when Mehr, Scannell, and Winner [9] tried to replicate Tsay’s [8] work and tested the robustness and the generalizability of Tsay’s findings (the precedence of visual over auditory cues in music judgment), they concluded that the previous findings were not robust enough since minor changes in methods generated significantly different or even opposite results. For example, Mehr et al. note that when presenting stimuli in pairings rather than triads, participants were unable to reliably identify a winner, while in triads they were able to. They suggest this is due to probabilities related with guessing–for example, if one can rule out one of three presentations, they can perform at 50% chance while guessing. In Brimhall’s [10] experiment, however, participants who observed the audiovisual stimulus provided similar ratings to those who experienced the audio-only clips, indicating an insignificant effect due to presentation conditions (audio-only, or audiovisual) and suggesting that visual stimulus did not influence the evaluation of music. Therefore, there is evidence that vision may not triumph as the dominant sense, and it is possible to judge the musical ratings without expecting the influence of visual feedback.

### 1.2. Visual Elements

Movements, or body gestures of performers during music performances are often considered as the most obvious area of study for visual elements that affect music evaluations. Trevor and Huron [11] tested the effect of performer movement on judgments of performance quality. To test this, the movements were created for animated stick figure performers who were performing both slow and faster passages that had either magnified, original, or diminished performance motion; participants adjusted the range of motion to create the best musical performance [11]. Participants significantly amplified the motions of the performers for the fast passages, while preferring only about normal movement for the lyrical passages. This indicates that greater performance motion exhibit superior performances (particularly with fast passages); perhaps the audience feels less inclined to increase the motion for lyrical or slower passages since it is already fairly expressive compared to inexpressive fast passages that have more technical demands which makes it difficult to add expressive motions. 

Researchers have also been interested in the relationship between body movements and musical expressivity and have explored the effect of non-verbal body gestures on the expressivity [12,13,14] and emotional quality [15] of music performances. They assigned the participants into visual-only, audio-only, or audiovisual groups and presented solo performances with one of three expressive manners: restrained, normal, or exaggerated intention to rate expressivity and emotional qualities [15,16]. Expressive intention had its greatest impact when the performances could be seen (visual-only), which reveals that not only is vision a useful source of information about manner, but it also specifies manner more clearly than the other groups [15,16]. Hence, there is a need to consider visual as well as sound information in music perception as the most effective factor that determines expressivity and emotions being conveyed. 

Weiss, Nusseck, and Spahn [16] also analyzed the same question as Trevor and Huron [13] examining the influence of ancillary gesture, but in clarinetists specifically. Participants viewed and rated videos of kinematic displays of clarinetists with optical markers attached to specific body parts to provide a full body recording of four different motion types on five general aspects of music—expressiveness, match of the movements to the music, musical fluency, professionalism, and overall impression of the performance [16]. Highest ratings were given to those who performed with predominant motion, and the lowest ratings were given for performances with overall low motion [16]. Authors propounded that this might have occurred because of the relationship between the perceived degree of motion and the intended level of expressiveness of a musician; a musician’s aim to purposely exaggerate the motion behaviour can enhance the perception of the expressivity and performance. As such, we have evidence that bodily gestures and movements enhance audiences’, and adjudicators’, experience and evaluations of musical performances.

### 1.3. Evaluators’ Musical Ability

Marozeau, Innes-Brown, Grayden, Burkitt, and Blamey [17] and Griffiths and Reay [18] focused on the effect of visual cues on music evaluations and whether evaluators’ musical training mediates this effect by asking musicians and non-musicians to rate: the difficulty of separating a four-note repeating melody from interleaved random distracter notes [13], and four video clips (professional/good audio; PA + amateur/bad visual; AV, PV+AA, PA+PV, AA+AV) on three musical aspects [18]. Marozeau et al. [17] found out that when there was no visual cue, musicians generally rated the melody segregation as less difficult than non-musicians, but when a visual cue was present, difficulty ratings for musicians and non-musicians were very similar, indicating that the effect of evaluators’ level of musical experience on their judgments of music is still unclear, and visual cues affect listeners’ perception of music. However, Griffiths and Reay [18] noticed evidence that visual information has a greater impact than auditory information on evaluations of performance quality, as the clip with bad audio + good video was rated significantly higher than that with good audio + bad video on all three evaluation measures, but also resulted in no significant effect of musical training on any of the evaluation measures. This result is in contrast to Marozeau et al.’s [17] findings and highlights a possible unimportance of evaluators’ musical ability.

Mitchell and MacDonald [19] explored the importance of visual or audio priming in identifying music performers, and which cue is stronger. Musicians were assigned into either: visual-audio; V-A (watched the target performer then listened to a line-up of target and distractors), or audio-visual; A-V (opposite to visual-audio order) and guessed the target performer [19]. While all participants identified the target above chance level regardless of the presentation order and the number of distractors, V-A’s rates were significantly higher than A-V’s indicating that although both audio and visual cues provide enough info to achieve the task, the findings presumably arose from the visual cues being more robust information when identifying performers and people being more sensitive to them than auditory cues [19]. Thus, the music industry should be aware that visual priming is more important than audio priming to correctly identify the targets.

## 2. Materials and Methods

All recruitment and data collection packages were evaluated by the Research Ethics Board at UNB Saint John and are on file as REB #004-2020. All stimuli and data are publicly available on the Open Science Framework at https://osf.io/qtv6j/. This project was a direct replication of the study by Tsay [3] and as such all experimental elements were designed to be as close as possible to the original.

### 2.1. Participants

Participants were recruited from the general public through distribution of a survey link via email and social media channels. Only individuals with little or no musical training were eligible to participate in this study. Participants were compensated with a $10 electronic gift card from Amazon. 

All studies in the SCORE project are designed to have 90% power to detect an effect size that is 50% as large as in the original study (with alpha = 0.05). The observed effect size in Tsay (2014) was d = 0.668. In order to have 90% power to detect d = 0.334, it was determined that 96 participants were required. The 96 participants that completed the study had a mean age of 28.5 years (SD = 9.0), and consisted of 67 female, 26 male, and 3 non-binary participants. 

### 2.2. Materials

The videoclips used were the same ones used by Tsay [3]. These consisted of three sets of eight clips, each of which had been extracted from elite international musical ensemble competitions. Each clip was six seconds long. As per the original study and the claim being tested by the replication, all clips were presented as visual only.

### 2.3. Procedure

Data collection was completed on the Gorilla online platform (https://gorilla.sc/). After completing a consent form, participants were asked for basic demographic information, including their level of musical training. Those participants who rated their level of musical training as ‘moderate’ or ‘professional’ were not allowed to proceed, while those with ‘little’ or ‘no’ musical training proceeded to the experimental task. While we have no way to guarantee that participants were fully honest about their level of musical training, we expect that participants would not have any motivation to mislead the experiment. Participants had no prior knowledge of what would be required to proceed to the experimental task, and as such we expect that the majority of participants would be honest. 

Participants were presented with the three clips from a set, after which they were asked to indicate which of the three performances should win a competition between the groups. The clips within a set were presented in a random order, and the order of sets was also randomized. Upon completion of all eight clips, participants saw a thank you message with an explanation of the reason for this task before being redirected to provide their email address if they wished to receive an Amazon gift card. 

## 3. Results and Discussion

**Figure 1:**
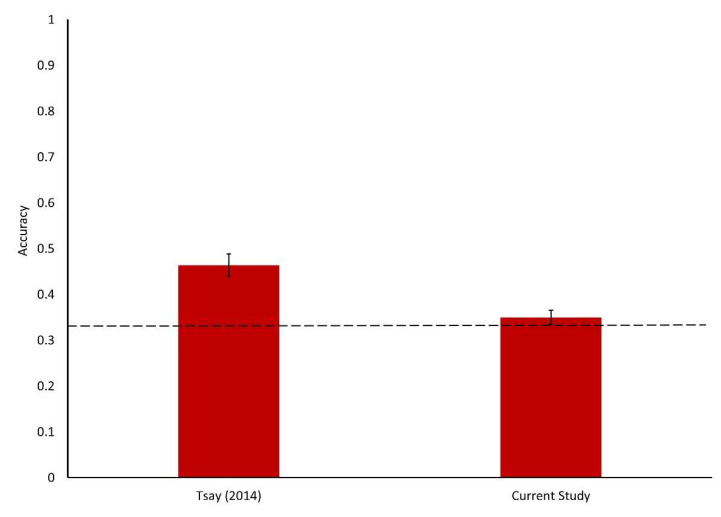
Means and standard errors for accuracy of assessment for Tsay (2014) and the current study. Dashed line indicates chance performance (0.333).

Participant accuracy was tabulated, with a mean score of 0.350 (SD = 0.147). Data were analyzed using a one sample t-test. As the task involved a 3-alternative forced choice task, the test value used was 0.333, which was indicative of chance performance. The t-test did not reveal a significant difference between the mean accuracy score and chance performance, t(95) = 1.151, *p* = 0.253, d = 0.117 [−0.084, 0.318] (See Figure 1). A supplementary analysis involved a Bayesian one sample t-test, which revealed a BF_01_ = 4.671. This suggests moderate support for the null hypothesis, which in this instance is that participants were unable to choose the winner of the contests at a rate better than chance.

In the original study by Tsay [3], the visual only condition was found to have an accuracy rate of 46.4%, which was significantly better than chance. However, in this direct, well-powered replication, we observed a null effect. Additionally, a Bayesian analysis suggests that our data provide moderate evidence in support of the null hypothesis. Given that this replication was conducted under the auspices of the SCORE project, and as such had high power, we argue that these findings should be given precedence over the original findings. However, it is also important for additional conceptual replications to be performed, in order to shed further light on the phenomenon of failure of thin slicing in the assessment of musical performances. For example, research could examine whether musical experts could reliably assess performances from these short, 6-**s** clips, and the modality conditions under which they would be able to do so. Another possibility would be to assess whether using longer musical excerpts would enable musical novices to complete the task successfully.

Previous research on effects of visual stimuli on rating of musical performances has shown that there are some reliable relationships. For example, in a study of non-musicians evaluating cello performances, participants used both auditory and visual cues to assess the performances [20]. Specifically, participants were unable to reliably assess quality of performance based on visual information alone, which is in alignment with the current findings. While there is no doubt that visual information can provide some cues toward the evaluation of a musical performance, it is also important to keep in mind that these cues are often secondary characteristics of the musical performance itself. For example, lengthy gesture of a violin bow may be indicative of very fluid performance, but the auditory musical information itself is equally indicative of that performance. In this study, however, we find that non-musicians are unable to reliably predict competition winners based on visual information, particularly from short clips. 

Some previous research suggests that non-musicians are able to successfully assess musical performances from visual information only [17,18]. However, in this replication study we find that this was not the case. Performance on the musical performance assessment task was found to be no different from chance performance. This suggests that those without musical expertise are unable to assess musical performances using such short video clips.

In understanding the impact of these findings on hiring practices in the music industry, Goldin and Rouse [21] examined whether discrimination in nonobjective aspects such as sex, race, or ethnicity apart from objective criteria (musical performance, resume content) exists in the hiring process of musicians by gathering audition records from the late 1950s to 1995. The authors found that the use of a screen increases the probability a woman will be advanced and hired, and blind auditions increase by 30% the proportion of females among new hires, suggesting that the blind auditions strengthened impartiality in hiring and increased the proportion of women in the period of the 1950s to 1995. These results clearly support an impact of discrimination on hiring in the music industry, and promote the adoption of the screen and blind auditions to help female musicians in their search for orchestral positions.

## 4. Conclusions

This study was conducted as a component of the SCORE project [4], which is an effort to increase confidence in research through conducting high-quality, well-powered direct and conceptual replications of existing research. In this instance, the claim being tested was not found to replicate successfully, suggesting that the original result may have been a false positive finding. Future study should seek to disambiguate these findings by conducting additional replications, possibly including a greater number of experimental trials in addition to a large number of participants. However, for now, the findings indicate that non-musicians are not able to predict the winner of a musical contest from 6-**s** long clips, based on visual information only.

## Data Availability

All data are available on Open Science Framework at https://osf.io/qtv6j.
